# Antisense Oligonucleotides (ASOs) in Motor Neuron Diseases: A Road to Cure in Light and Shade

**DOI:** 10.3390/ijms25094809

**Published:** 2024-04-28

**Authors:** Silvia Cantara, Giorgia Simoncelli, Claudia Ricci

**Affiliations:** 1 Department of Medical, Surgical and Neurological Sciences, University of Siena, 53100 Siena, Italy; cantara@unisi.it; 2 Unit of Neurology and Clinical Neurophysiology, Department of Neurological and Motor Sciences, Azienda Ospedaliero-Universitaria Senese, 53100 Siena, Italy; simoncelligiorgia1@gmail.com

**Keywords:** antisense oligonucleotides (ASOs), motor neuron diseases, spinal muscular atrophy, amyotrophic lateral sclerosis, spinal and bulbar muscular atrophy, innovative therapy, nusinersen, tofersen, clinical trials

## Abstract

Antisense oligonucleotides (ASOs) are short oligodeoxynucleotides designed to bind to specific regions of target mRNA. ASOs can modulate pre-mRNA splicing, increase levels of functional proteins, and decrease levels of toxic proteins. ASOs are being developed for the treatment of motor neuron diseases (MNDs), including spinal muscular atrophy (SMA), amyotrophic lateral sclerosis (ALS) and spinal and bulbar muscular atrophy (SBMA). The biggest success has been the ASO known as nusinersen, the first effective therapy for SMA, able to improve symptoms and slow disease progression. Another success is tofersen, an ASO designed to treat ALS patients with *SOD1* gene mutations. Both ASOs have been approved by the FDA and EMA. On the other hand, ASO treatment in ALS patients with the *C9orf72* gene mutation did not show any improvement in disease progression. The aim of this review is to provide an up-to-date overview of ASO research in MNDs, from preclinical studies to clinical trials and, where available, regulatory approval. We highlight the successes and failures, underline the strengths and limitations of the current ASO research, and suggest possible approaches that could lead to more effective treatments.

## 1. Introduction

In the landscape of modern medicine, the advent of molecular therapeutics has propelled the field towards unprecedented levels of precision and specificity. Among the groundbreaking innovations, antisense oligonucleotides (ASOs) have emerged as a class of therapeutic agents with transformative potential, offering a unique avenue for targeted genetic modulation [1,2,3]. These oligonucleotides, typically 15–25 bases in length, are designed to selectively modify protein synthesis by complementary binding to specific regions of target mRNA [1], steering away from the conventional paradigm of protein-focused therapeutics.

To fully understand the potential of ASOs, an exploration of their intricate composition is essential. Beyond their nucleotide sequence, ASOs undergo chemical modifications strategically incorporated into their backbone. Phosphorothioate linkages, 2′-O-methyl groups, and locked nucleic acids (LNAs) are among the key modifications. In phosphorothioate linkage, a sulphur atom replaces one of the non-bridging oxygen atoms in the phosphate group, enhancing stability and resistance to nuclease degradation [4]. The 2′-O-methyl groups refer to a chemical modification at the 2′ position of the ribose (or deoxyribose) sugar in nucleic acids in which a methyl group (-CH3) is added. This modification is preferred for RNA as it helps overcome some of the limitations associated with the natural susceptibility of RNA to degradation and affects the RNA’s interaction with cellular machinery and proteins [5]. In LNAs, the ribose ring is chemically constrained by a methylene bridge connecting the 2′-oxygen and the 4′-carbon of the ribose, creating a “locked” structure [5]. By incorporating LNAs into the ASO sequence, the resulting LNA-ASO exhibits increased stability, improved binding affinity, and enhanced resistance to nuclease degradation compared to traditional oligonucleotides. Additional modifications contributing to improved ASO pharmacokinetics include peptide nucleic acids (PNAs), replacing the sugar-phosphate backbone with a peptide-like structure [6]; GalNAc conjugation, facilitating liver targeting [7]; and hydrophobic modifications, enhancing cellular uptake and distribution [8]. These tailored molecular architectures ensure the longevity of ASOs in physiological environments and facilitate their efficient delivery to target cells, which are critical for therapeutic efficacy (Table 1).

**Figure 1:**
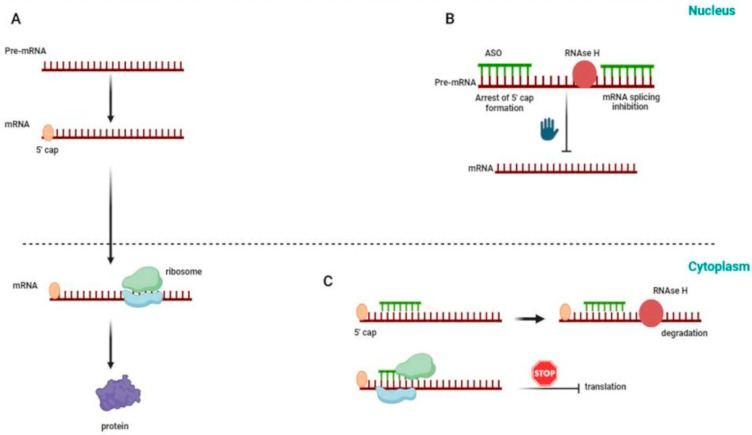
Mechanisms of ASO action in the regulation of gene expression: (**A**) Normal gene expression in the absence of ASO; (**B**) ASO can enter the nucleus and induce both 5′ cap formation and RNAse H-mediated mRNA splicing; (**C**) In the cytoplasm, ASO can either interfere with ribosome assembly or activate RNAse H-induced mRNA degradation due to ASO-mRNA heteroduplex formation.

**Figure 2:**
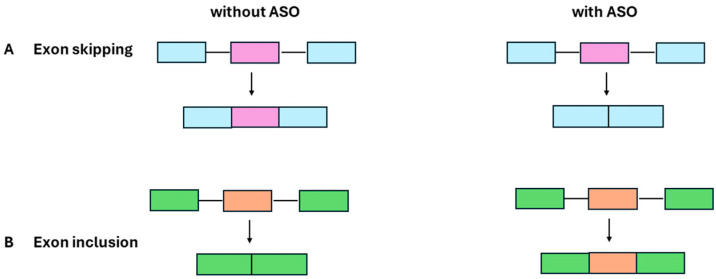
Mechanisms of ASO action in the regulation of RNA processing: (**A**) Exon skipping without or with ASO and (**B**) exon inclusion without or with ASO therapy.

The adaptability of ASOs stems from their ability to engage with target mRNA through precise Watson-Crick base pairing [9]. This interaction sets the stage for a multitude of mechanisms through which ASOs can exert their therapeutic effects. One paradigmatic mechanism involves the recruitment of cellular machinery, such as the endonuclease RNase H, triggering mRNA cleavage and subsequent degradation. This approach is particularly powerful when the therapeutic objective is to attenuate the expression of deleterious proteins associated with genetic disorders. However, the influence of ASOs extends beyond mere mRNA degradation (Figure 1). Steric hindrance, another aspect of their mechanism, enables ASOs to interfere with the splicing process, dictating the inclusion or exclusion of specific exons [10] (Figure 2). This modulation of gene expression holds promise in conditions where aberrant splicing events underlie the pathophysiology, such as certain types of muscular dystrophy.

In this paper we reviewed the use of ASOs as therapeutic agents in motor neuron diseases such as spinal muscular atrophy, amyotrophic lateral sclerosis, and spinal bulbar muscular atrophy, summarizing their mode of action and clinical trials.

## 2. Overcoming CNS Delivery Challenges: Strategies for Antisense Oligonucleotide Administration

The administration and delivery of ASOs in central nervous system (CNS) disorders are particularly challenging due to the presence of the blood–brain barrier (BBB), a highly selective semi-permeable layer of endothelial cells that acts as a filter. The chemical properties of ASOs, such as negative charge, high molecular weight, and hydrophilicity, prevent diffusion across the BBB and reduce the efficacy of systemic administration. To overcome these problems, intrathecal (IT) or intraventricular injection has been used to deliver ASOs directly into the CNS, bypassing the BBB. Following the injection of single-stranded phosphorothioate- and 2′-MOE-modified ASOs into the cerebrospinal fluid (CSF), rapid distribution throughout the spinal cord and to most regions of the brain is observed [11,12,13,14,15,16]. Approximately 20% of the injected dose remains in brain tissue with a peak 4–6 h after injection, while 80% or more is found in the systemic circulation [12,15] following normal CSF turnover pathways. In the case of 2′-MOE-modified oligonucleotides, the half-life in CNS tissues ranges from 3 weeks to 6 months, while the effects of single-stranded phosphorothioate ASOs on gene expression can last from 6 weeks to more than 6 months after a single injection, depending on the chemical design and method of administration [13,15,17]. The longest-lasting effects are achieved with 2′-MOE-modified oligonucleotides, where all internucleosidic linkages are phosphorothioate, combined with intracerebroventricular bolus injection as the method of delivery to the CNS. After intrathecal administration, the highest ASO concentrations are found near the injection site in the spinal cord, with lower concentrations in other regions of the spinal cord and cortical regions of the brain [12,15,18,19]. A similar distribution is found after intracerebral ventricular administration, but in this case, higher drug concentrations are found in the tissue surrounding the ventricle [20].

Given the invasiveness of this procedure, several approaches have been considered to deliver ASOs to the CNS. Intranasal administration, using the olfactory and trigeminal nerve pathways, can deliver drugs to the CNS [21,22,23,24]. In addition, the use of delivery particles has been shown to improve ASO transport into the CNS after systemic administration. Glucose-coated polymeric nanocarriers allow efficient brain accumulation of ASOs by non-invasive intravenous administration, presumably due to their multivalent binding to glucose transporter 1 expressed on the plasma membrane of brain capillary endothelial cells, with the highest brain accumulation shown when their size is less than 50 nm and the number and density of glucose are approximately 50 per 100 block copolymer strands on their surface [25]. Peptide-conjugated ASOs are another type of delivery particle that can bypass the blood–brain barrier. In a spinal muscular atrophy (SMA) disease model, these ASOs were present in the brain (cortex, brainstem, cerebellum), spinal cord, peripheral skeletal muscle, and liver after intravenous administration and were able to rescue the phenotype and dramatically extend the lifespan of severe SMA mice without significant side effects [26], making this approach very promising.

## 3. Motor Neuron Diseases

Motor neuron diseases (MNDs) are a group of sporadic and inherited neurodegenerative disorders that result in the total or predominant loss of motor neurons. Upper motor neurons are located in the primary motor cortex of the brain, and their axons connect to the brainstem (corticobulbar tract) and to the corticospinal tract of the spinal cord (corticospinal tract). Lower motor neurons are located in motor nuclei in the brainstem or in the anterior grey matter of the spinal cord. They are responsible for transmitting the signal from the upper motor neuron to the effector muscle, as their axons connect to the muscles of the limbs and bulbar region. Degeneration of motor neurons leads to loss of voluntary muscle function. Depending on which muscles are affected, patients with MNDs develop muscle weakness and atrophy, bulbar involvement, and respiratory failure.

MNDs generally include spinal muscular atrophy (SMA), amyotrophic lateral sclerosis (ALS), and spinal and bulbar muscular atrophy (SBMA).

## 4. Spinal Muscular Atrophy (SMA)

Spinal muscular atrophy (SMA) is an autosomal-recessive neuromuscular disease characterised by the progressive degeneration of alpha motor neurons in the anterior horn of the spinal cord, resulting in muscle atrophy and loss of muscle function [26]. Its incidence ranges from 1:6000 to 1:11,000 live births in the general population [27,28,29]. SMA is caused by deletions or point mutations in the *SMN1* gene, which encodes the survival motor neuron (SMN) protein. SMN plays a key role in the proper function and survival of motor neurons. Complete loss of the SMN protein is lethal at the embryonic stage [30]. Based on the age of onset and the severity of clinical symptoms, which is inversely related to the amount of SMN protein available at the motor neuron level, SMA is classified into different phenotypes (i.e., SMA type 0, type 1, type 2, type 3, and type 4) [31]. The severity of the disease depends, at least in part, on the number of copies of a second gene, called *SMN2*, which is a centromeric copy of *SMN1* that arose from a duplication event during primate evolution [32]. The *SMN2* gene is almost identical to *SMN1*, but the transition of a C to a T in exon 7 inactivates a splicing enhancer and simultaneously introduces an exonic splicing silencer, resulting in abnormal mRNAs lacking exon 7 [33,34,35,36,37]. These transcripts lacking exon 7 produce very low levels of SMN protein because they are unstable and therefore degraded rapidly and cannot compensate for the loss of SMN protein caused by *SMN1* gene deletions/mutations. There is a variable number of copies of the *SMN1* and *SMN2* genes in the human population, including individuals with more than two copies of *SMN2*, with no known deleterious effect. Patients with type 1 SMA typically have two copies of the *SMN2* gene, patients with type 2 SMA have three copies of the *SMN2* gene, and patients with type 3 SMA have three to four copies of this gene. These observations support the idea that the number of copies of *SMN2* is a robust modifier of disease [38,39,40,41], suggesting that increasing the amount of SMN protein should have therapeutic effects in SMA.

**Figure 3:**
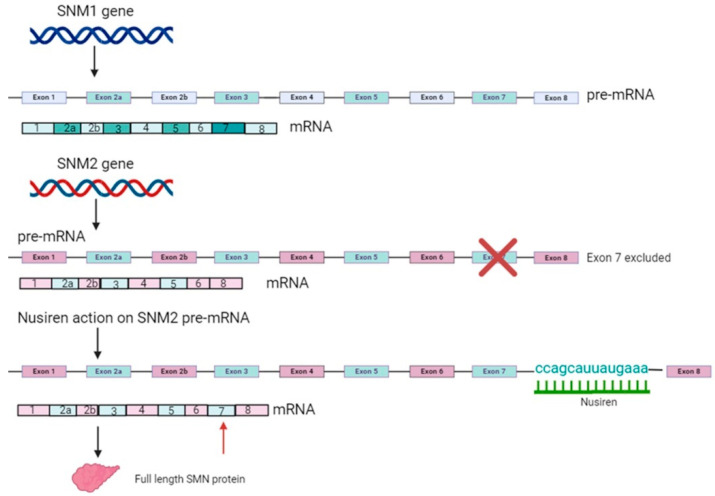
Nusinersen restoration of the SNM2 gene function: The *SNM1* gene leads to a full-length transcript resulting in a functional protein. The *SNM2* gene, on the contrary, results in a non-functional protein due to the skipping of exon 7. Nusinersen is able to restore a full-length protein by acting on pre-mRNA and avoiding the exclusion of exon 7.

Various ASOs have been designed to block intronic splicing silencers or induce splicing enhancers to prevent exon 7 skipping [42,43,44]. Nusinersen (trade name Spinraza) was the first SMA orphan drug approved by the Food and Drug Administration (FDA) and by the European Medicines Agency (EMA) for the treatment of SMA in children and adults. It is a 2′-MOE-modified ASO designed to increase SMN protein expression by modulating the splicing of the *SMN2* precursor messenger RNA (pre-mRNA) to restore a full-length mature messenger RNA (mRNA) from the *SMN2* pre-mRNA. Nusinersen targets a specific splicing silencer site (ISS-N1, intronic splice silencing) in intron 7 of *SMN2* and prevents the binding of specific splicing repressors, hnRNPA1 and hnRNPA2, to ISS-N1, allowing the integration of exon 7 into the final transcript and increasing the synthesis of a full-length functional SMN protein [45,46] (Figure 3).

As ASOs do not cross the blood–brain barrier, nusinersen must be administered intrathecally. A phase 1 clinical trial in which increasing doses of nusinersen were administered by lumbar puncture to children with type 2 and type 3 SMA showed that this ASO was well tolerated [47]. The phase 1 study was followed by two different open-label phase 2 studies, one in the same population as the phase 1 study and the second in infantile-onset SMA infants. The first showed an increase in survival in nusinersen-treated patients compared with sham-treated patients [12,48]. In addition, nusinersen-treated infants showed an improvement in motor function scores that was never observed in the natural history of type 1 SMA patients [48]. Clinical trials in patients with type 2 SMA also showed improvement in motor function compared to a decline or no change in sham-treated patients [49]. These results led to the approval of nusinersen by the FDA in 2016 and by the EMA in 2017 for the treatment of all types of SMA, just five years to the day after the first patient was exposed to the drug [50]. Table 2 summarises the main steps of this pathway, while Table 3 reports nusinersen clinical trials.

A Phase 2 open-label, single-arm study (NURTURE—NCT02386553) is underway to evaluate the effects of nusinersen in infants with a genetic diagnosis of SMA (most likely to develop type 1 or type 2 SMA), where treatment is initiated before the onset of symptoms [52,53]. The main objective of the study is to determine whether nusinersen can prevent or delay the onset of the disease and/or result in a milder form of the disease (primary endpoint: time to death or respiratory intervention; secondary endpoints: attainment of motor milestones, change from baseline in growth parameters, proportion of participants developing clinically manifested SMA at 13 and 24 months of age). Since infants with a genetic diagnosis of SMA, with one functional copy of the *SMN1* gene and two or three copies of the *SMN2* gene, are expected to develop severe or fatal symptoms in the first years of life, an internal control group was considered unnecessary and ethically unjustifiable. The study is expected to be completed in early 2025.

In addition to nusinersen, a Phase 1 trial (NCT05575011) is currently underway for BIIB115, an ASO with an N-methylacetamide (NMA)-modified chemical backbone that improves efficacy and provides the opportunity to assess patient outcomes with long-interval dosing.

Nusinersen is a clear example of how it is feasible to implement collaborative strategies among different stakeholders (e.g., research centres, industry, and regulators) to build a robust pathway based on robust preclinical, translational, and clinical evidence to support the regulatory process [31]. In this way, it is possible to provide patients with effective therapies that can improve symptoms and slow disease progression, even in neurodegenerative diseases.

## 5. Amyotrophic Lateral Sclerosis (ALS)

ALS is a fatal neurodegenerative disease characterized by the progressive degeneration of motor neurons in the motor cortex, brainstem, and spinal cord, with consequent atrophy of voluntary muscles [54]. The incidence of ALS is approximately 1–2.6 cases per 100,000 person-years, and the average survival time from onset to death is 3–4 years [55]. There is no cure for ALS. The only approved drugs, riluzole and edaravone, provide only modest survival benefits and are often associated with multiple side effects [56,57]. Most cases of ALS occur sporadically, with no reported family history, whereas approximately 10% are familial (fALS), with autosomal dominant inheritance in most cases [58]. Pathogenic variants have been identified in more than 30 genes [59]. However, in approximately 70% of patients with familial ALS, the disease is associated with variants in four genes: chromosome 9 open reading frame 72 (*C9orf72*), superoxide dismutase 1 (*SOD1*), TAR DNA binding protein (*TARDBP*), and fused in sarcoma (*FUS*) [60].

Although a unique cellular pathway associated with mutations in these genes has not been identified, several studies have shown that the majority of the monogenic causes of ALS act through a toxic gain of function of the mutated gene [61]. In these cases, ASO therapies that directly modify the disease-causing genes and neutralise the toxic gene products hold great promise. At present, there are antisense therapies at various stages of development that directly target the transcripts of *SOD1*, *C9orf72*, and *FUS*.

### 5.1. SOD1 Gene

The first gene identified as a cause of ALS was *SOD1* [62], which accounts for about 20% of familial ALS cases and up to 2% of sporadic cases [63]. More than 200 different mutations in this gene have been associated with ALS (see https://alsod.ac.uk/, accessed on 25 April 2024). Although the exact pathogenic mechanism of *SOD1* mutations is not fully understood, several lines of evidence suggest that it is associated with a toxic gain of function that impairs neuronal function and survival [64]. Reducing the expression of toxic SOD1 proteins in rodent models of ALS has been shown to delay disease onset and increase survival [16,65,66,67].

Based on these observations, the first ASO therapy targeting *SOD1* mRNA via an RNase H1 mechanism of action was developed [16]. ASO 333611, a 2′-MOE gapmer, produced a dose-dependent reduction in *SOD1* mRNA and protein in transgenic rats expressing human mutant *SOD1^G93A^* RNA, with a 40–60% reduction in the brainstem and spinal cord [16]. The reduction in SOD1 expression was well tolerated, delayed disease onset, and prolonged survival by 37% after disease onset in the transgenic rats. These encouraging preclinical data prompted a phase 1, double-blind, placebo-controlled clinical trial (NCT01041222) to evaluate the safety and tolerability of intrathecally delivered ASO 333611 in SOD1 ALS patients [68], making ASO 333611 the first experimental antisense drug to be administered intrathecally to patients for the treatment of a neurodegenerative disease. Given its novelty, a single course (12 h slow intrathecal infusion) of a low dose (0.15 to 3 mg) was used. No drug-related safety issues were observed in the study. Although no reduction of SOD1 protein levels in cerebrospinal fluid was observed, this study was a landmark for ASO therapy, demonstrating for the first time that intrathecal infusion of ASOs is safe in humans and effective in animals, and establishing a protocol to rapidly advance ASOs from initial selection into clinical trials.

Meanwhile, advances in ASO therapies for other neurological diseases, including SMA, identified more effective designs to improve the efficacy and tolerability of these drugs. As a result, the ASO approach to ALS was subsequently redesigned to incorporate more advanced technologies. The next-generation ASO, BIIB067 (IONIS-SOD1Rx, then called tofersen, trade name Qalsody), is a 2′-MOE mixed backbone ASO that was identified following an oligonucleotide screen in cell culture and *hSOD1^G93A^* transgenic mice and rats [19]. BIIB067, which targets a different region of the *SOD1* pre-mRNA and is designed for the treatment of ALS patients carrying mutations in the *SOD1* gene (Figure 3), was shown to be approximately six times more potent than ASO 333611 in cultured cells and three to four times more potent in inhibiting *SOD1* mRNA expression in transgenic rodents. In addition, administration of BIIB067 to transgenic *hSOD1^G93A^* rodents prior to disease onset significantly prolonged survival, slowed motor impairment, and reduced neuromuscular damage [19]. ASO therapy also reduced serum levels of phosphorylated neurofilament heavy chain (pNFH) [19], a cytoskeletal protein released in cerebrospinal fluid during axonal injury and correlated with disease severity [69]. When administered after disease onset, ASOs suppressed further increases in pNFH and restored neuromuscular activity close to baseline. Intrathecal injection of BIIB067 into non-human primates (NHPs) resulted in a dose-dependent reduction of *SOD1* mRNA and protein in CNS tissues and CSF.

Based on the strong functional recovery observed in rodent models and the favourable pharmacokinetics in NHPs, a randomised, double-blind, placebo-controlled Phase I/II study (VALOR; NCT02623699) was initiated to evaluate the tolerability and pharmacokinetics of intrathecal administration of tofersen in familial ALS patients carrying a mutation in the *SOD1* gene [70]. Single and multiple ascending doses were used. This and subsequent ASO trials used an intrathecal bolus rather than the intrathecal catheter and external pump employed in the first *SOD1* ASO trial. Tofersen was generally well tolerated and safe. The highest concentration of tofersen was more effective in reducing CSF SOD1 levels than lower doses. There was also a trend towards a slowing of the decline in ALSFRS-R measured in the highest dose group, particularly in the fast-progressing subgroup. Levels of plasma and CSF pNFH and neurofilament light chain (NfL) were also reduced in this group.

In light of these promising results, tofersen was advanced to a 28-week, randomised, double-blind, placebo-controlled Phase 3 trial (VALOR; NCT02623699) using the maximum dose from the previous study. Given the analysis from the tofersen Phase I/II trial, and to reduce the impact of the known heterogeneity of the patients, participants were divided into fast- and slow-progressing subgroups, with the effect on progression in the fast-progressing group as the primary outcome. This study showed that, after 28 weeks of treatment with tofersen, cerebrospinal fluid SOD1 and plasma neurofilament light chain levels were reduced, but clinical endpoints were not reached [71]. However, several secondary and exploratory endpoints supported favourable clinical and biomarker trends for tofersen treatment. In particular, in the fast-progressing group, tofersen reduced CSF SOD1 and plasma NfL by 38% and 67%, respectively, and showed a benefit in respiratory (slow vital capacity) and muscle strength. Slow progressors exhibited similar but more limited reductions in SOD1 (26%) and NfL (48%) and very small reductions in ALSFRS-R, slow vital capacity, and muscle strength. Based on these results, the FDA approved tofersen in April 2023 for the treatment of patients with amyotrophic lateral sclerosis associated with a mutation in the *SOD1* gene, followed by the EMA’s Committee for Human Medicines (CHMP) approval in February 2024. A long-term, open-label Phase 3 extension study (NCT03070119) is underway to follow participants who received tofersen and assess long-term safety, tolerability, and efficacy.

**Figure 4:**
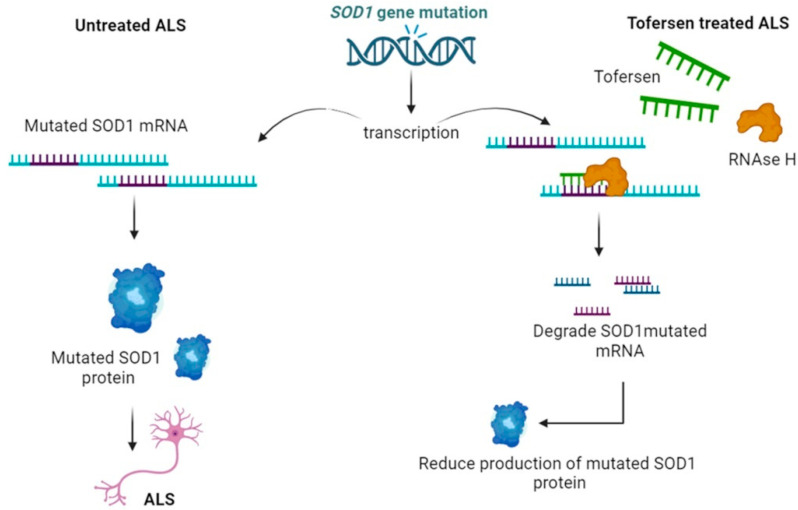
Tofersen mechanism of action in ALS: Mutated *SOD1* leads to mutated SOD1 protein which develops into ALS. Tofersen, through an RNAse H mechanism, degrades *SOD1* mutated mRNA, thus reducing the production of mutated SOD1 protein.

Figure 4 summarises the mechanism of action of tofersen.

A recent study evaluating the effects of tofersen treatment in patients with *SOD1*-ALS in a “real-world setting” (a 12-month multicentre cohort study from the German Early Access Program) confirmed an effective therapeutic approach with a reduction in serum NfL levels, but also demonstrated a reduction in CSF pNFH. The therapy was safe, as no persistent symptoms were observed. Pleocytosis, increased protein levels, and intrathecal immunoglobulin synthesis were common CSF findings that need to be evaluated in future studies [72].

Based on the favourable safety and therapeutic outcomes for ALS patients, the SOD1-ASO is now being considered for the treatment of asymptomatic gene carriers in a new trial (ATLAS, NCT04856982). The hypothesis to be tested is that treating individuals with SOD1-ASO at the first evidence of biomarker changes, but before clinical evidence of motor neuron disease, may slow or prevent the onset of the disease. This randomised, placebo-controlled trial aims to recruit ~150 pre-symptomatic carriers without clinically manifest ALS (from May 2021 to August 2027). Participants will be monitored frequently for serum NfL levels. If a participant shows an increase in NfL, they will be randomised to receive either 100 mg tofersen or placebo every month for the following 2 years (1:1 to tofersen or placebo). The study endpoints will be the proportion of participants who develop clinically manifest ALS within 12 months and 24 months of randomisation and the time from randomisation to the development of clinically manifest ALS. An open-label extension period will allow participants who develop clinical ALS to receive tofersen in the open-label arms of the study [73]. In Table 4, we summarised preclinical studies, and in Table 5, clinical studies of ASOs targeting *SOD1*.

### 5.2. C9orf72 Gene

The expanded GGGGCC hexanucleotide repeat in intron 1 of the *C9orf72* gene is the most common genetic cause of frontotemporal dementia (FTD) and ALS, accounting for ~40% of fALS cases and 5–7% of sALS cases [74]. The *C9orf72* gene contains between 2 and 30 repeats in healthy individuals. In ALS patients, however, the number of repeats can reach hundreds or thousands [75,76]. Transcription of this gene results in three main transcripts (V1, V2, V3). The repeat region is located in the first intron of transcripts V1 and V3, whereas the V2 transcript contains the repeat in its promoter region. V1 encodes a short isoform of the C9orf72 protein, while V3 and V2 encode the same long isoform but contain different untranslated first exons (1a and 1b, respectively) [77].

The process by which *C9orf72* expansion leads to disease is not fully understood. Several mechanisms have been suggested: bidirectional transcription of the mutant *C9orf72* gene, with expression of sense and antisense RNA strands that can form RNA foci and fold the GGGGCC repeats into a G-quadruplex capable of sequestering important RNA-binding proteins and chromatin modifiers [78,79,80]; production of dipeptide repeat strings (DPRs) due to a non-canonical, repeat-associated, non-ATG-mediated (RAN) translation mechanism that alters cellular proteostasis [80,81,82]; haploinsufficiency of *C9orf72*, which limits its physiological functions (vesicle trafficking, autophagy, lysosomal processing, and immune response) [83,84]. It is likely that both gain- and loss-of-function mechanisms are responsible for the development of the disease [84,85]. Therefore, ASO therapies should mitigate the toxic gain of function resulting from the repeat expansions while maintaining the levels of the normal allele. This will reduce the toxicity of DPRs and repeat-containing RNA without suppressing physiological protein functions.

A unique opportunity for targeting C9orf72 arises from the multiple transcript variants produced. By targeting C9orf72 transcript variants 1 and 3, which carry the expansion, it is possible to reduce the expression of transcripts containing the expansion while preserving variant 2 and thus C9orf72 protein levels.

The studies performed in vitro and in animal models showed promising results. The use of ASOs was effective in reducing RNA foci in iPSC-derived neurons, and administration to transgenic mice ameliorated behavioural defects [85,86]. In addition, in a single patient carrying mutant *C9orf72*, CSF levels of polyGP dipeptide repeats, a stable biomarker of C9orf72-ALS [87], decreased following multiple intrathecal injections of ASOs. The procedure was well tolerated, demonstrating for the first time the possibility of using ASOs in the clinic for C9orf72 ALS [88].

The first clinical trial was conducted to assess the safety and tolerability of the ASO BIIB078, a phosphorothioate backbone ASO, which selectively targets *C9orf72* transcript variants 1 and 3 carrying the expansion, inducing RNAse H degradation, for the treatment of adult ALS patients with *C9orf72* expansion (NCT03626012). In this randomised, placebo-controlled Phase I clinical trial, 114 participants with C9orf72-ALS (excluding fast progressors) were recruited to receive ascending doses of BIIB078 or placebo by intrathecal infusion. BIIB078 was well tolerated but did not meet any of the endpoints (change in ALSFRS-R, slow vital capacity, and muscle strength) [89]. Moreover, patients receiving BIIB078 showed a trend toward greater clinical decline and increased levels of NfL in plasma, and further development of BIIB078 was discontinued.

A randomised, placebo-controlled Phase I/II clinical trial (NCT04931862) was also initiated to evaluate the safety, tolerability, and efficacy of the variant-selective, stereopure, phosphoryl guanidine backbone C9orf72-lowering ASO WVE-004 [90,91], in patients with ALS and/or FTD due to *C9orf72* expansion. This ASO targets a sequence near the exon 1b–intron junction, yielding more durable RNase H-mediated knockdown. Again, treatment with WVE-004 failed to show clinical benefit after 24 weeks, leading Wave to discontinue further development. Although a 48% reduction in CSF polyGP was observed, this was not associated with stabilisation or improvement in functional outcomes compared to placebo (https://www.globenewswire.com/news-release/2023/05/23/2674200/0/en/Wave-Life-Sciences-Announces-Topline-Results-from-Phase-1b-2a-FOCUS-C9-Study-of-WVE-004-for-C9orf72-associated-Amyotrophic-Lateral-Sclerosis-and-Frontotemporal-Dementia.html, accessed on 25 April 2024). Given that the *C9orf72* DNA containing the expansion is transcribed bidirectionally, producing both sense and antisense RNA strands that form RNA foci, and that all currently used ASOs bind and degrade only the sense strand, leaving DPRs and RNA foci produced by antisense *C9orf72* unaffected, it is hypothesised that the failure of these trials is due to their inability to neutralise antisense strands [89]. In this view, the results of these studies may provide valuable information that will lead to a deeper understanding of this form of ALS, which is clearly much more complex than previously thought.

An Investigational New Drug Application (IND) approved by the FDA (IND141673) is currently underway to test the mixed backbone ASO afinersen in a single subject, a 60-year-old man with the *C9orf72* expansion [88]. Afinersen targets the intronic region flanking the GGGGCC repeat expansion. It suppresses the expression of V1 and V3 while allowing basal levels of V2. Prior to this application, toxicological studies in rodents, sheep, and monkeys supported the safety of afinersen [88]. Due to the chemical modification pattern used, no delivery vehicle was required, and the therapeutic was injected directly into the patient’s spinal fluid to target the cells of interest. At the start of treatment, the patient had mild motor changes with elevated polyGP in the CSF. Eight escalating doses were administered intrathecally over 60 weeks, starting in August 2019. Afinersen was well distributed throughout the CSF, and compared to baseline, CSF polyGP levels decreased by 80%, indicating that this ASO is active and reduces C9orf72 expansion consequences. ALSFRS-R remained stable throughout treatment. At the time of this review, there are no registered clinical trials for afinersen.

Table 6 summarises preclinical studies, and Table 7 clinical studies of ASOs targeting *C9orf72*.

### 5.3. FUS Gene

Mutations in the *FUS* gene are associated with a rare and aggressive form of ALS, often with juvenile onset [61]. FUS is an RNA-binding protein that plays a key role in RNA metabolism, including regulation of splicing and translation of mRNA, and in DNA repair. To fulfil its role, FUS requires frequent nuclear/cytoplasmic translocation [94]. Mutations in the *FUS* gene are associated with cytoplasmic mislocalisation of the FUS protein, which forms cytoplasmic inclusions that are associated with neuronal degeneration [95]. Therefore, the pathogenetic mechanisms associated with *FUS* mutations appear to be due to loss of function in the nucleus and gain of toxic functions in the cytoplasm [96].

**Figure 5:**
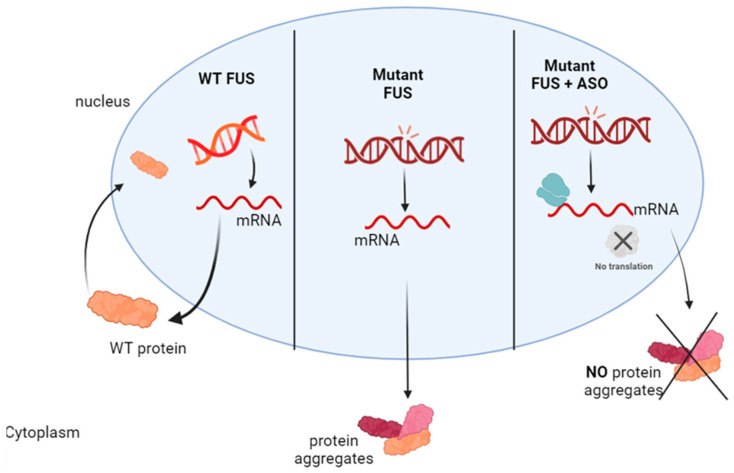
Mechanism of action of ASO in FUS-ALS: Mutated *FUS* leads to cytoplasmic mislocalisation of the FUS protein and formation of protein aggregates in the cytoplasm. ASO therapy, decreasing the expression of both FUS mutant and wild-type transcripts, reduces FUS-containing aggregates.

As *FUS* mutations are relatively rare, studies targeting them are limited. However, recent preclinical studies showed that the non-allele-specific FUS ASO ION363 (also called jacifusen), which targets intron 6 of the *FUS* gene, reduced the expression of both FUSP525L mutant and wild-type transcripts in the brain and spinal cord of a mouse model of FUS-ALS. ION363 also reduced levels of insoluble FUS protein and insoluble RNA-binding proteins found in FUS aggregates (Figure 5). In addition, ION363 prevented neurodegeneration of lumbar motoneurons and loss of neuromuscular junction innervation [97]. These results motivated the use of ION363 in a patient with the P525L mutation in FUS in a compassionate use IND application [97]. This application did not require toxicology studies in rodents or monkeys. The patient, a 26-year-old woman, received monthly ascending doses of 20 mg to a maximum of 120 mg intrathecally between June 2019 and March 2020. Treatment started six months after disease onset, when the disease was already at an advanced stage. The participant did not experience any serious adverse events. The patient died of ALS in May 2020. Neuropathological examination showed that ION363 reduced wild-type and mutant FUS protein with a decrease in FUS aggregates. There was little nuclear FUS staining in the spinal cord and motor cortex, and FUS-containing aggregates in motor neurons were reduced compared with the untreated ALS-FUSP525L control, in which FUS aggregates were abundant [97].

Based on these positive results, the clinical efficacy, safety, and pharmacology of ION363 (jacifusen) are being evaluated in Phase III clinical trials in ALS patients with *FUS* mutations (FUSION; NCT04768972). A total of 64 patients will be enrolled by March 2024 and will be randomised in a 2:1 ratio to receive jacifusen or placebo monthly or bimonthly for 29 weeks in part 1 of the study. Part 2 of the study will be a subsequent 72-week open-label extension period in which all patients will receive jacifusen. Participants will be transferred to the Part 2/open-label extension of the study if they show significant functional decline during Part 1. The primary outcome includes an assessment of the ALSFRS-R, the time to rescue, and the survival time without ventilator support. Secondary outcomes will be the evaluation of muscle and lung function, survival, and changes in CSF FUS protein and neurofilaments. Results are expected in June 2025.

### 5.4. Other ALS-Related Genes

An interesting target for ALS therapy could be TDP-43, encoded by the *TARDBP* gene. It is a DNA/RNA-binding protein that plays a key role in RNA processing, regulating transcription, splicing, mRNA transport, mRNA stabilisation, and miRNA maturation [98]. Under normal conditions, TDP-43 is mainly located in the nucleus, but in many neurodegenerative diseases such as ALS, TDP-43 is sequestered in the cytoplasm, where it forms characteristic inclusions [99]. Nuclear depletion of TDP-43 leads to detrimental changes in the cell [98,100]. Given its fundamental role, an approach involving the total downregulation of TDP-43 levels must be ruled out [101,102,103]. An alternative approach is to attempt to correct the splicing defects of specific mRNAs that result from nuclear depletion of TDP-43. One interesting potential target is stathmin-2. Stathmin-2, encoded by the *STMN2* gene, is a microtubule-binding protein that is abundant in motor neurons and critical for axonal stability and regeneration. Its expression is reduced in both sporadic and familial ALS patients [104]. TDP-43 binds *STMN2* pre-mRNA and represses the inclusion of a cryptic exon in the mRNA. When TDP-43 is depleted, this cryptic exon is incorporated into the mRNA, resulting in a truncated, non-functional transcript. The subsequent decrease in the functional level of stathmin-2 appears to contribute to the pathogenesis of ALS [105]. An ASO that sterically blocks the cryptic splice site region of the stathmin-2 pre-mRNA, similar to the action of TDP-43, was able to restore normal splicing and functionality of stathmin-2 in human motor neurons and in a mouse model [106]. An ASO with a similar mechanism of action, called QRL-201, is being investigated in a randomised, double-blind, placebo-controlled Phase 1 study to assess its safety and tolerability in ALS (ANQUR, NCT05633459).

Another approach to developing ASO therapies is to consider modifier genes as potential targets. In this case, the target is not a causative gene, but a relatively common gene variant that confers an increasing risk of ALS. This strategy may be an important alternative for ALS patients without known mutations, mainly those with sporadic ALS. Ataxin-2 is a well-characterised example of such a modifier. It is an RNA-binding protein found in RNA-containing granules, encoded by the *ATXN2* gene. Ataxin-2 has a polyglutamine tract encoded by cytosine-adenine-guanine (CAG) repeats, which are less than 30 in healthy individuals. A number of CAG repeats of 34 or more is associated with the severe neurodegenerative disease spinocerebellar ataxia 2 (SCA2) [107], while intermediate CAG expansions (27–33 glutamines) are associated with an increased risk of ALS [108,109]. Treatment of a TDP-43 mouse model with an ASO that reduces ataxin-2 levels has been shown to have a beneficial effect on motor function and survival [108]. A placebo-controlled Phase 1/2 study was initiated in September 2020 to evaluate the safety and pharmacokinetics of the intrathecally administered BIIB105, an ASO designed to reduce ataxin-2 levels in ALS patients with or without intermediate CAG expansions (ALSpire; NCT04494256). The study is expected to end in July 2026.

## 6. Spinal Bulbar Muscular Atrophy (SBMA)

Spinal and bulbar muscular atrophy (SBMA), also known as Kennedy disease, is an X-linked recessive neuromuscular disease characterised primarily by the degeneration of lower motor neurons [110,111]. The disease typically affects only males, although females can be carriers and sometimes experience muscle cramps. The prevalence is 1–2 per 100,000 people [111,112]. Onset usually occurs at around 30–40 years of age, with a range of 18–64 years [113]. The disease is characterised by the progressive degeneration of muscle and lower motor neurons, resulting in muscle weakness, atrophy, and fasciculations. The progression is slow compared to other motor neuron diseases, with a decline in muscle strength of about 2% per year [114]. As the bulbar muscles become affected, people with SBMA develop difficulties with speech and swallowing [111].

SBMA is caused by a CAG repeat expansion in the androgen receptor (*AR*) gene on the X chromosome, with a corresponding increase in the length of a polyglutamine tract in the AR protein [115]. Notably, SBMA was the first of many neurological disorders caused by expanded polyglutamine tracts to be identified. In healthy individuals, the repeat is present with a range of 9–36 CAGs, whereas in SBMA patients it is expanded to 39–72 CAGs, with the repeat length correlating with age of onset and disease severity [115].

The AR is a ligand-dependent transcription factor that, through its N-terminal domain, interacts with coregulatory proteins to regulate the transcription of androgen-responsive target genes. Mutation of the *AR* gene causes both gain and loss of function of the receptor. However, the primary effect of the mutation is to alter the protein structure such that the receptor becomes toxic to motor neurons and muscle, causing a toxic gain of function of the protein. The loss-of-function effect results in partial androgen insensitivity with gynecomastia and reduced fertility, which does not appear to be directly related to the progressive weakness and loss of motor neurons [116]. The mechanisms underlying the neurodegenerative gain of function in SBMA are not fully understood [117]. Nuclear accumulation and aggregation of polyQ-ARs may contribute to motor neuron degeneration through a variety of molecular mechanisms involving disruption of multiple processes such as transcriptional regulation, protein homeostasis, intracellular trafficking, mitochondrial function, and cellular signalling [118].

Preclinical studies have shown that ASOs can mediate the degradation of mutant *AR* transcripts, reducing mRNA and protein levels in animal models. ASOs used in this case are gapmers that bind to AR mRNA and trigger RNase H cleavage and RNA degradation. A first study focused on two different 2′,4′-constrained ethyl (cEt) gapmers that caused a dose-dependent reduction of *AR* mRNA in HUVEC cells, named ASO1 and ASO2, and investigated whether they were able to ameliorate peripheral muscle pathology in transgenic SBMA mouse models [119]. ASO1 targets a region of the *AR* mRNA that is conserved between human and mouse transcripts, whereas ASO2 targets a human-specific region. ASO1 and ASO2 were administered subcutaneously to the AR113Q and humanised BAC fxAR121 SBMA mouse models, respectively. ASO treatment resulted in a significant knockdown of *AR* mRNA and an almost complete reduction of AR protein levels in the mouse quadriceps muscle, with an overall improvement in the disease phenotype. As the gapmers used in this study are unable to cross the blood–brain barrier, AR expression in the spinal cord was unaffected, demonstrating that skeletal muscle plays an important role in SBMA and can be considered as a target for ASO treatments.

A subsequent study used intracerebroventricular administration of ASOs in SBMA mice to evaluate the effect of knocking down AR transcripts in neurons rather than in muscle [120]. The AR-97Q mouse model, which expresses both murine and transgenic human AR protein, was treated with intracerebroventricular injection of either ASO-AR1 (targeting both human and murine *AR*) or ASO-2 (mouse-specific), both of which are 2′-MOE gapmers. Treatment resulted in a significant decrease in mutant AR mRNA and protein in the spinal cord and brain, while AR levels in peripheral muscle were unaffected. Mice treated with either ASO also showed a marked improvement in the clinical phenotype, which was confirmed by immunohistochemical analysis showing numerous markers of improvement, such as reduced neuronal degeneration and improved neuromuscular junction endplate maturation. Despite the negligible uptake of ASO into skeletal muscle, the muscles of ASO-AR1-treated mice showed restored fibre size and reduced atrophy compared to controls. The results of these preclinical studies are summarised in Table 8.

Taken together, these studies show that ASO treatment could be effective in SBMA and that AR knockdown in both peripheral tissues and the CNS is associated with an improved clinical phenotype of the disease. However, as both groups used gapmers that are unable to cross the blood–brain barrier, the treatments were mutually exclusive of either the CNS or peripheral muscles, depending on the route of administration. Given recent advances in the identification of nanocarriers and apoptotic bodies that can enhance oligonucleotide blood–brain barrier penetration [25,121,122], the next generation of treatments for SBMA may consist of ASOs able to target both CNS and peripheral tissues with a single injection.

## 7. Discussion and Future Directions

In recent years, the use of ASOs has given new impetus to research and the development of effective therapies for many previously untreatable diseases. These include motor neuron diseases, i.e., sporadic and inherited neurodegenerative conditions that entirely or predominantly injure motor neurons and include SBMA, spinal muscular atrophy (SMA), and amyotrophic lateral sclerosis (ALS). In this field, the use of ASOs has led to both great successes and failures. The greatest success was certainly the development of a therapy for SMA based on an ASO known as nusinersen, which is able to compensate for the lack of the SMN protein in SMA by influencing the splice efficiency of exon 7 in the *SMN2* gene. The drug has been shown to significantly improve motor function and increase survival in SMA patients. In just a few years, it has been possible to move from the pre-clinical phase to the approval and marketing of the drug (FDA in 2016 and EMA in 2017) thanks to effective collaboration among different stakeholders to build a robust pathway based on pre-clinical, translational, and clinical evidence to support the regulatory process. Nusinersen has been the first effective therapy for SMA, demonstrating that it is possible to provide patients with effective therapies that can improve symptoms and slow disease progression. This has paved the way for new therapeutic approaches.

Another very positive result was obtained in the treatment of ALS associated with mutations in the *SOD1* gene. Tofersen is an ASO designed for the treatment of ALS patients carrying mutations in the *SOD1* gene. The data collected so far have shown that it reduces biomarker degeneration levels (NfL) and can slow disease progression, especially if treatment is started early after the onset of symptoms. Further long-term clinical studies are still ongoing, but the FDA approved tofersen in 2023 under its Accelerated Approval Program, which allows early approval of drugs that treat serious conditions and fill an unmet medical need based on surrogate markers, and EMA approval arrived in February 2024. This is a milestone in the history of ALS research that has shown for the first time that the disease, at least in some of its forms, can be a treatable condition.

These results have stimulated the search for ASO-based therapies for ALS associated with mutations in other genes. Clinical trials with ASOs targeting *FUS*, *STMN2*, and *ATXN2* are underway and will show whether these therapies are effective in the coming years. The case of hexanucleotide expansion of the *C9orf72* gene deserves a separate discussion. Given the frequency of this mutation in both familial and sporadic forms of ALS, ASOs directed against the sense mRNA, targeting the mRNA containing the repeat expansion, have been developed and tested with encouraging results in animal models. Based on the promising data, two different companies started phase 1/2 clinical trials with ASOs targeting different regions of the sense repeat mRNA (WVE-004-NCT04931862, Wave Life Sciences; BIIB078-NCT03626012, Biogen/Ionis). Unfortunately, both trials were halted after an interim analysis of results as neither the primary nor secondary endpoints were met.

At this stage, it is not clear what the cause of these failures might be. Various hypotheses have been suggested [123,124]. The ASOs could affect the expression level of wild-type *C9orf72*. These ASOs were designed not to affect the expression of the normal C9orf72 protein, but an effect on global C9orf72 expression in patients could explain the negative effects, as previously observed in mice [125]. In addition, the antisense repeat mRNA may be more important than predicted. This antisense RNA and the DPRs translated from it are not affected by the ASOs tested, and it remains to be clarified whether degradation of the sense RNA affects the antisense RNA. Finally, the question of why ASOs that reduce the amount of sense repeat RNA and the DPRs translated from it appear to have a negative rather than neutral effect on ALS patients remains unanswered.

The positive and negative results obtained so far highlight some important general aspects of the development of ASO-based therapies. First, when considering the treatment of genetic diseases with ASOs, the genetic background/inheritance pattern of the disease must be taken into account when evaluating possible therapeutic approaches.

The first distinction is between two main disease mechanisms caused by pathogenic variants: loss of function (LoF) and gain of function (GoF). Autosomal recessive disorders are usually associated with LoF variants. LoF variants can lead to loss of the gene product, nonsense-mediated decay of RNA transcripts, production of unstable proteins or proteins with no or reduced function [126]. Regardless of the mechanism of action, the general therapeutic approach to LoF is to attempt to restore protein function by resetting the reading frame to either the canonical transcript or a modified transcript that produces a (at least partially) functional protein [127]. In the case of SMA, the presence of the paralogue gene *SMN2* partially facilitated this process, as it was possible to target a specific splicing silencer site in intron 7 of *SMN2* and prevent specific splicing repressors from binding to it, allowing the integration of exon 7 into the final transcript and increasing the synthesis of a full-length functional SMN protein.

In general, LoF variants are relatively easier to deal with than GoF variants. LoF genes are often approached by restoring healthy copies, whereas GoF genes are approached by targeting the diseased allele, gene, or protein for degradation. Examples of such toxic effects include, but are not limited to, missense variants and expanded repeats, which confer additional functions to the protein, increasing its propensity to form aggregates, a common feature of several neurodegenerative diseases. Therefore, one way to develop an effective therapy might be to degrade toxic aggregates or prevent their formation. This approach could stop or slow the progression of the disease, but in principle could also prevent disease manifestation if the treatment is started before the onset of the disease. ASOs could play a key role in such a therapeutic strategy. In this case, there are two different options. A selective strategy involves the use of ASOs that degrade the toxic (mutant) variant while preserving the wild-type form of the protein. The subtle differences between wild-type and pathogenic alleles could be exploited to selectively target the mutant (pre-)mRNA and thus selectively prevent the expression of only the toxic protein variant [128,129]. On the other hand, non-selective targeting of the affected gene could reduce both the toxic and wild-type variants, thus reducing the function of the protein. This is certainly a simpler approach and does not require the use of tailored therapies (different ASOs for different mutations), but it implies that physiologically necessary functions of the wild-type protein are also reduced. A non-specific knockdown of a protein can have serious, unpredictable consequences, so it is extremely important to assess the extent to which the gene can tolerate downregulation without causing additional damage. These negative effects on the wild-type protein could be the reason for the failure of ASO treatment in ALS patients with the *C9orf72* gene mutation, as discussed above. Such negative effects have not been seen with tofersen treatment in ALS patients with *SOD1* mutations. However, long-term follow-up of these patients is needed, as the observation that recessive null mutations are associated with early and progressive neurological dysfunction [130] suggests that continued suppression of the normal *SOD1* allele may have adverse effects.

Another important aspect to consider is the need for a thorough understanding of the molecular mechanisms underlying the disease (particularly in the presence of alterations in the different genes associated with ALS). This would make it possible to define within what margins it is possible to act, and what can and cannot be done. Research, even basic research, still has a fundamental role to play in this field. This can also be of relevance for the development of more complex therapeutic approaches. One example is the attempt to correct splice defects due to nuclear depletion of TDP-43. The major advantage of this strategy is that a much larger population of ALS patients than just mutation carriers could benefit from this therapeutic strategy, as mislocalisation of TDP-43 is seen in almost all ALS patients. However, in view of its fundamental role, a total downregulation of TDP-43 levels must be ruled out. An alternative approach is to attempt to correct the splicing defects of specific mRNAs that result from nuclear depletion of TDP-43. However, since many mRNAs are aberrantly processed in the absence of nuclear TDP-43, it is unclear whether correcting these single, specific splicing abnormalities will prove effective. It is possible that several of these mis-spliced mRNAs may need to be corrected with a cocktail of ASOs before any therapeutic benefit is achieved. Alternatively, it may be helpful to identify other players that play a key role in the TDP-43-mediated splicing process. SYF2 is a pre-mRNA splicing factor that is recruited to the spliceosome to regulate splicing. When downregulated, it reverses TDP-43 pathology and improves TDP-43 function, including RNA processing, in preclinical models [131]. Thus, ASO-mediated downregulation of SYF2 could restore mis-splicing of multiple mRNAs. To find out whether this strategy also works in patients with ALS, clinical trials will be crucial.

**Figure 6:**
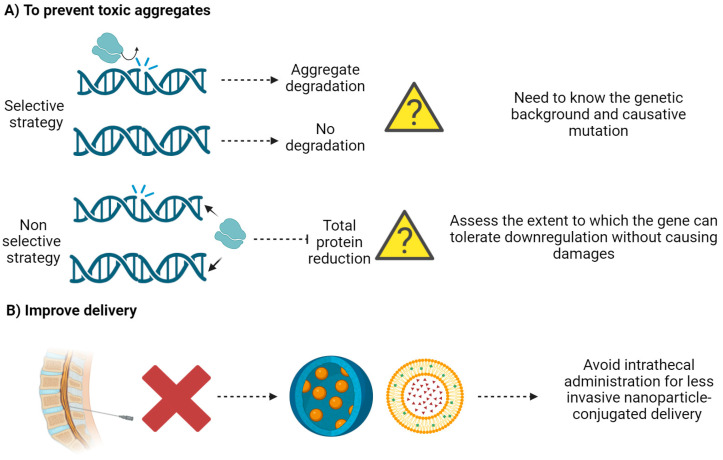
ASO research presents several still open issues. (**A**) To develop an effective therapy, ASOs could be useful in toxic aggregate degradation. By applying a selective strategy, ASOs can degrade the toxic (mutant) variant while preserving the wild-type form of the protein. Alternatively, a non-selective targeting of the affected gene may be chosen, degrading both the toxic and wild-type variants, thus reducing the whole function of the protein. In the first case, the pathogenetic mutation must be known; in the second approach, it is extremely important to assess the extent to which the gene can tolerate downregulation without causing additional damage. (**B**) Commonly, ASOs are intrathecally administered. This represents an invasive procedure that can be replaced by developing nanoparticle-conjugated ASOs.

Currently, ASOs are delivered by intrathecal administration, a rather invasive and technically demanding procedure. Although commonly used in the clinical setting, the invasiveness and cost of the procedure is stimulating the development of alternative routes of drug delivery. Advances in chemistry to enhance potency and conjugation of targeting ligands to the ASO are being developed to provide more effective antisense drugs. The use of delivery particles, such as glucose-coated polymeric nanocarriers and peptide-conjugated ASOs, is very promising for the future as they can cross the BBB and may enhance ASO transport into the CNS after systemic administration. However, delivery by nanoparticles may show toxicity linked to the nature of nanoparticles used. For example, protein-based nanoparticles can exert cardiotoxicity, hepatotoxicity, and hypersensitivity; metal-based nanoparticles show anxiogenic effects together with genotoxicity; and lipid-based nanoparticles exhibit cardiopulmonary distress and hypersensitivity [132] (Figure 6).

Another important point to consider is when to start treatment with ASOs. By the time the first symptoms of the disease appear, the motor neurons have already suffered significant damage that cannot be eliminated. Therefore, therapy can only slow down or at best stop the progression of the disease. Two clinical trials in pre-symptomatic individuals with a genetic diagnosis of SMA (NURTURE, NCT023865539) and carriers of mutations in the *SOD1* gene (ATLAS, NCT04856982) are underway to shed light on this issue. If the results are positive, we could see a revolutionary change for some MNDs, from incurable and fatal to not only treatable but also preventable.

In light of these new perspectives, genetic screening for SMA and SOD1-mediated ALS is of paramount importance. In the case of SMA, newborn screening (NBS) allows the immediate initiation of specific treatment for children with SMA to halt irreversible motor neuron loss and disease progression and ensure motor development like that of children without the neuromuscular disease. The Advisory Committee on Heritable Disorders in Newborns and Children (ACHDNC) added NBS for SMA to the Recommended Uniform Screening Panel in July 2018, and thanks to national or regional pilot projects, SMA NBS was implemented in several countries [133]. About ALS, considering that mutations in the *SOD1* gene account for about 20% of fALS patients and up to 2% of sALS cases, prompt screening for *SOD1* mutations should be performed in all new ALS patients with both familial and sporadic presentations.

In summary, ASO therapy has made remarkable progress in recent years, bringing significant benefits to the treatment of motor neuron diseases. The greatest success has been the development of nusinersen, the first effective therapy for SMA approved by the FDA and EMA, able to improve symptoms and slow disease progression. This was followed a few years later by tofersen, which was approved to treat ALS patients with SOD1 mutations. On the other hand, there is still a long way to go regarding other forms of ALS associated with mutations in other genes, particularly *C9orf72*. A deeper understanding of the pathogenetic mechanisms linked to the presence of mutations, together with the development of increasingly effective and high-performance molecules, may make it possible to develop new therapies against these neurodegenerative diseases.

## Figures and Tables

**Table 1:** Main ASO modifications with advantages and disadvantages.

Name of Modification	Type of Modification	Advantages	Disadvantages
Single-stranded phosphorothioate	Replacement of one of the non-bridging oxygen atoms in the phosphate backbone with a sulphur atom	Improved nuclease resistance in plasma, tissues, and cells	Cytotoxicity when delivered at high concentrations due to non-specific binding with certain proteins
2′-O-Methoxyethyl (2′-MOE)-modification	Modifications at the 2′ position of the sugar moiety	Enhanced nuclease resistance, lower cell toxicity, and increased binding affinity	Impaired RNase H cleavage of the complementary RNA
2′-MOE gapmers	A central core of phosphorothioate-modified DNA is flanked by 2′-MOE-modified RNA bases	Induces RNase H cleavage, increases binding affinity to the target, mitigates non-specific cleavage	Immunogenic reaction still possible
2′-O-[2-(methylthio)ethyl] or 2′-O-MTE modification	Modifications at the 2′ position of the sugar moiety	Improved binding to human serum albumin, high binding affinity to target RNA	Limited resistance to exonuclease degradation
Phosphoryl guanidine backbone modification	Phosphoryl guanidine modification of the phosphate group at internucleotidic positions	Increased nuclease resistance, enhanced affinity and selectivity to target sites, enhances exon skipping	Reduced cellular uptake
Mixed-backbone oligonucleotides (MBOs)	Alternative phosphorothioate and phosphodiester linkages in the 2′-O-methylribonucleosides	Improved affinity to RNA, RNase H activation, better pharmacological and pharmacokinetic properties	The efficiency of gene silencing can vary depending on target mRNA secondary structure, accessibility, and cellular context. This variability may lead to unpredictable outcomes and require optimization for each specific target.
Locked nucleic aacids (LNA)	The ribose ring is chemically constrained by a methylene bridge connecting the 2′-oxygen and the 4′-carbon of the ribose, creating a “locked” structure	Increased binding affinity, enzymatic stability	Increased liver toxicity

**Table 2:** Summary of nusinersen preclinical studies.

Year	Results	Ref.
2006	Identification of the ISS-N1 sequence within *SMN2* intron 7 Synthesis of the first complementary ASO	[46]
2008	Synthesis of ASO 10–27 with high affinity to ISS-N1 First preclinical studies on an SMA mouse model	[45]
2010–2011	Improved SMN protein expression following administration of ASO 10–27 by intrathecal or intracerebroventricular injection in SMA mice Ameliorated disease phenotype No increase in lifespan of mice	[14]
2011	Amelioration of peripheral symptoms after subcutaneous injection of ASO 10–27 in SMA mice Improved lifespan by more than 25-fold	[51]
2011	Adequate distribution at the level of the spinal cord after intrathecal injection in non-human primates No significant side effects.	[14]

**Table 3:** Summary of Nusinersen clinical trials.

Phase	Type of Study	SMA Type	n° of Patients	Administration (Doses)	Clinical Outcomes	Ref.
I (NCT01494701) (NCT01780246)	Open-label	2/3	28	Intrathecal bolus injection (1, 3, 6, 9 mg)	Improved HFMSE scores in the 9 mg groups post-dose	[47]
II (NCT01839656)	Open-label	1	20	Intrathecal injection (6 mg and 12 mg equivalents)	Increased improvement in HINE-2 and CHOP-INTEND test assessments	[12]
III (ENDEAR NCT02193074)	Double-blind, randomised, and sham-controlled	1	121	Intrathecal injection (12 mg equivalents)	Higher percentage of motor-milestone response and higher percentage of CHOP-INTEND response compared to control group	[48]
III (CHERISH NCT02292537)	Double-blinded, multicentre and sham-controlled	later-onset SMA (2–12 years)	126	Intrathecal injection (12 mg)	Significant improvement in motor function compared to control group (increase from baseline to month 15 in the HFMSE score of at least 3 points)	[49]
II (NURTURE NCT02386553)	Open-label single-arm	1/2 presymptomatic	25	Intrathecal injection	Underway	[52,53]

HFMSE, Hammersmith Functional Motor Scale-Expanded; HINE-2, Hammersmith Infant Neurological Exam-Part 2; CHOP-INTEND, Children’s Hospital of Philadelphia Infant Test of Neuromuscular Disorders.

**Table 4:** Summary of preclinical studies of ASOs targeting *SOD1*.

Year	Results	Ref.
2006	ASO 333611 produced a dose-dependent reduction of *SOD1* mRNA and protein in *SOD1^G93A^* rats, delayed disease onset, and prolonged survival by 37% after the onset. The reduction in SOD1 expression was well tolerated.	[16]
2018	BIIB067 was more potent than ASO 333611 in inhibiting *SOD1* mRNA expression in cultured cells and in transgenic rodents. BIIB067 administration to transgenic *SOD1^G93A^* rodents before disease onset significantly prolonged survival, slowed motor impairment, and reduced neuromuscular damage. ASO therapy reduced serum levels of pNFH.	[19]

pNFH: phosphorylated neurofilament heavy chain.

**Table 5:** Summary of clinical trial of ASOs targeting *SOD1*.

Phase	Type of Study	n° of Patients	Administration (Doses)	Clinical Outcome	Ref.
I (NCT01041222) ASO 333611	double-blind, placebo-controlled	22	A single course (12 h slow intrathecal infusion) of a low dose (0.15 to 3 mg)	No drug-related safety issues. No reduction of SOD1 protein levels in CSF	[68]
I/II (VALOR NCT02623699) BIIB067 (Tofersen)	Randomised, double-blind, placebo-controlled trial	50	Intrathecal injection (20, 40, 60, or 100 mg)	Tofersen was generally well tolerated and safe. The highest concentration was the most effective in reducing CSF SOD1 levels and slowed decline in ALSFRS-R	[70]
III (VALOR NCT02623699) BIIB067 (Tofersen)	Randomised, double-blind, placebo-controlled trial	108	Intrathecal injection (100 mg)	Reduction of CSF SOD1 and plasma neurofilament light chain levels after 28 weeks of treatment. Clinical endpoints were not reached. Several secondary and exploratory endpoints supported favourable clinical and biomarker trends, particularly in the fast-progressing group	[71]
III (NCT03070119) BIIB067 (Tofersen)	Long-term, open-label extension	138	Intrathecal injection (100 mg)	The aim is to assess long-term safety and tolerability of tofersen	[71]
III (ATLAS NCT04856982) BIIB067 (Tofersen)	Presymptomatic carrier	150 expected (2021–2027)	In case of an increse in NfL, the participant will be randomised to receive either 100 mg tofersen or placebo	The aim is to assess the effectiveness of tofersen in pre-symptomatic adult carriers of *SOD1* mutations with elevated neurofilament levels	[73]

CSF: cerebrospinal fluid.

**Table 6:** Summary of preclinical studies of ASOs targeting *C9orf72*.

Year	Results	Ref.
2013	Studies in patient-derived fibroblasts [92], iPSC neurons [85], or motor neurons [93] demonstrate that C9orf72-targeting ASOs could potently reduce repeat-containing C9orf72 transcripts and clear intranuclear RNA foci	[85,92,93]
2016	Intracerebroventricular injection of intron-targeting ASOs ameliorates behavioural defects in transgenic mice carrying a bacterial artificial chromosome with the full human repeat-containing C9orf72	[86]

**Table 7:** Summary of clinical studies of ASOs targeting C9orf72.

Phase	Type of Study	n° of Patients	Administration (Doses)	Clinical Outcome	Ref.
I (NCT03626012) BIIB078	Randomised, placebo-controlled trial	114 (no fast progressors)	Intrathecal infusion of ascending doses (10 to 90 mg)	No drug-related adverse events No change in ALSFRS-R, slow vital capacity, and muscle strength trend toward a greater decline in patients receiving the highest dosage	[89]
I/II (NCT04931862) WVE-004	Randomised, double-blind, placebo-controlled trial	35	Intrathecal injection (single dose of 10, 30, or 60 mg OR multiple doses of 10 mg either every four or 12 weeks)	Single and multiple doses were generally well tolerated. Reduced poly(GP) levels in the CSF. No significant clinical benefits observed after six months on any efficacy measure	[90,91]
IND (IND141673 (Afinersen)	Investigational New Drug Application	1 (mild motor changes/elevated polyGP in the CSF)	Intrathecal injection (Eight escalating doses from 0.5 mg/kg to 2.0 mg/kg)	Treatment safely tolerated. Good distribution throughout the CSF. CSF polydiGP levels reduced by approximately 80%. ALSFRS-R stable throughout treatment	[88]

**Table 8:** Summary of preclinical studies of ASO targeting *AR*.

Year	Results	Ref.
2014	Subcutaneous administration of: ASO1 (targeting a region of the *AR* mRNA conserved between human and mouse transcripts) to the AR113Q mouse model, and ASO2 (which targets a human-specific region of the *AR* mRNA) to the humanised BAC fxAR121 SBMA mouse model. Significant knockdown of *AR* mRNA and an almost complete reduction of AR protein levels in the quadriceps muscle, with an overall improvement in the disease phenotype. AR expression in the spinal cord unaffected.	[119]
2015	Intracerebroventricular injection of either ASO-AR1 (targeting both human and murine AR) or ASO-2 (mouse-specific) in the AR-97Q mouse model, which expresses both murine and transgenic human AR protein. Significant decrease in mutant *AR* mRNA and protein in the spinal cord and brain. AR levels in peripheral muscle unaffected. Marked improvement in the clinical phenotype, confirmed by immunohistochemical analysis, with restored fibre size and reduced atrophy also in the muscles.	[120]

## Data Availability

Not applicable.
